# Aspirin and cancer: has aspirin been overlooked as an adjuvant therapy?

**DOI:** 10.1038/bjc.2011.289

**Published:** 2011-08-16

**Authors:** R E Langley, S Burdett, J F Tierney, F Cafferty, M K B Parmar, G Venning

**Affiliations:** 1 MRC Clinical Trials Unit, London, UK; 2 Pharmaceutical Research Services, High Wycombe, UK

**Keywords:** aspirin, cyclooxygenase inhibition, randomised trials

## Abstract

Aspirin inhibits the enzyme cyclooxygenase (Cox), and there is a significant body of epidemiological evidence demonstrating that regular aspirin use is associated with a decreased incidence of developing cancer. Interest focussed on selective Cox-2 inhibitors both as cancer prevention agents and as therapeutic agents in patients with proven malignancy until concerns were raised about their toxicity profile. Aspirin has several additional mechanisms of action that may contribute to its anti-cancer effect. It also influences cellular processes such as apoptosis and angiogenesis that are crucial for the development and growth of malignancies. Evidence suggests that these effects can occur through Cox-independent pathways questioning the rationale of focussing on Cox-2 inhibition alone as an anti-cancer strategy. Randomised studies with aspirin primarily designed to prevent cardiovascular disease have demonstrated a reduction in cancer deaths with long-term follow-up. Concerns about toxicity, particularly serious haemorrhage, have limited the use of aspirin as a cancer prevention agent, but recent epidemiological evidence demonstrating regular aspirin use after a diagnosis of cancer improves outcomes suggests that it may have a role in the adjuvant setting where the risk:benefit ratio will be different.

There is a substantial body of epidemiological evidence indicating that regular use of aspirin or other traditional non-steroidal anti-inflammatory drugs (NSAIDs) is associated with a reduced risk of developing cancer (Bosetti *et al*, 2006; Cuzick *et al*, 2009). Selective cyclooxygenase-2 (Cox-2) inhibitors have a more precise molecular target compared with traditional NSAIDs and were designed to have a better safety profile in terms of gastrointestinal toxicity. Several studies started evaluating Cox-2 inhibitors both as prevention agents and as potential therapeutic agents for patients with established cancer until concerns about cardiovascular system toxicities were raised in 2004 (Bresalier *et al*, 2005) when most of this work was discontinued.

Aspirin inhibits both Cox-1 and Cox-2, although it preferentially inhibits Cox-1 (Simmons *et al*, 2004). The first indication of a possible role for aspirin in cancer therapy came in 1968 when Gasic *et al* (1968) showed that platelet reduction was associated with a 50% reduction in metastases in mice. This was followed by the demonstration that aspirin administration produced a significant reduction in metastases in mice (Gasic *et al*, 1972) and that it prevented osteolysis produced by bony metastases from carcinosarcoma cells in rats (Powles *et al*, 1973). These findings were not followed up in human clinical trials. After a 15-year interval, there were a number of epidemiological studies of cancer prevention but almost no work assessing aspirin as a potential therapeutic agent against cancer. Two recent epidemiological studies demonstrating that regular aspirin use *after* a cancer diagnosis improves outcomes suggest that aspirin could have a role as an adjuvant therapy in cancer (Chan *et al*, 2009; Holmes *et al*, 2010).

## Aspirin as an anti-cancer agent: mechanisms of action

### Cox inhibition

Aspirin inhibits the enzyme Cox; two isoforms Cox-1 and Cox-2 are well characterised (Simmons *et al*, 2004). Cox converts arachidonic acid to prostaglandin H2, which in turn produces biologically active prostaglandins that influence pathophysiological processes in a range of tissues including angiogenesis, apoptosis, cell proliferation and migration, inflammatory response and thrombosis (Simmons *et al*, 2004; Ulrich *et al*, 2006). Inhibition of prostaglandin synthesis is considered the predominant mechanism by which NSAIDs act as anti-inflammatory agents, but it is unclear whether the anti-cancer properties of these agents can be solely attributed to Cox inhibition.

Support for the hypothesis that the anti-cancer effects of NSAIDs result from prostaglandin inhibition include the observation that higher concentrations of prostaglandins are found in cancers compared with the surrounding normal tissues and led to the hypothesis that prostaglandins might accelerate the growth and invasion of cancer (Easty and Easty, 1976). Growth factors and oncogenes also induce prostaglandin synthesis (Levine, 1981). More recently, Cox-2 overexpression has been identified in a number of different malignancies and it has been hypothesised that Cox-2 prostaglandins promote tumourigenesis by inhibiting apoptosis, modulating the immune system and regulating tumour-associated angiogenesis (Cha and DuBois, 2007). Understanding the relative roles of Cox-1 and Cox-2 in tumour progression is complicated as the enzymes function both in the tumour and in the peri-tumour stromal environment. For example, in a mouse model using stromal fibroblasts, Cox-1 was required for a polyp to develop to 1 mm, and after that, Cox-2 induction and microsomal prostaglandin E2 were required for further growth (Takeda *et al*, 2003).

Mice modified genetically to be deficient in either Cox-1 or Cox-2 provide insights into the normal physiological functions of these enzymes and their possible role in carcinogenesis (Langenbach *et al*, 1999). Inducing a mutation in the *Apc* gene normally results in 100% of mice having intestinal neoplasia. In Cox-1 or Cox-2 deficient mice, the effect of this mutation is decreased by 80% indicating that inhibition of either Cox-1 or Cox-2 could be an effective anti-cancer strategy (Chulada *et al*, 2000). Similarly, in mouse skin cancer models, genetic or pharmacological inactivation of either Cox-1 or Cox-2 results in reduced tumourigenesis (Tiano *et al*, 2002). The half-life of aspirin in the human body is only 15–20 min; therefore, the clinical observations (Bosetti *et al*, 2006; Chan *et al*, 2009) that once-daily administrations of aspirin appear to have an anti-cancer effect, particularly in tumours that overexpress Cox-2 are intriguing. Patrono *et al* (2001) suggest that the key mechanistic feature is a persistent decrease in platelet Cox-1 activity leading to downregulation of Cox-2 in tumours or the peri-tumoural environment. Aspirin, unlike other NSAIDs, binds irreversibly to Cox, and the anucleate platelet is unable to re-synthesise the enzyme resulting in decreased thromboxane A2 and reduced platelet aggregation. In addition, it is hypothesised that platelets affect the development and spread of metastases through a number of mechanisms, including facilitating the adhesion of cancer cells to circulating leukocytes and endothelial cells, and permitting adhesion to the endothelium and transmigration. They also protect circulating cancer cells from immune-mediated clearance by natural killer cells, and produce growth factors that promote angiogenesis (Honn *et al*, 1992).

### Non-Cox-dependent pathways

**Figure 1:**
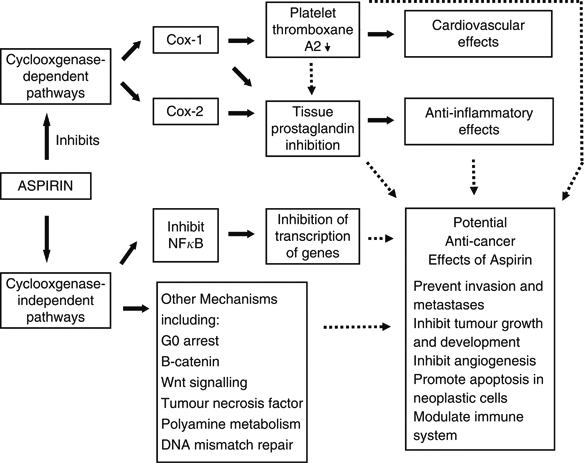
Aspirin mechanisms of action and pathophysiological effects. Black block arrows indicate known mechanisms. Dotted black arrows indicate potential mechanisms that could contribute to anti-cancer effects. Cox=cyclooxygenase; NFκB=nuclear factor-κB.

Several lines of evidence suggest that non-Cox-dependent pathways may also contribute to aspirin's anti-cancer effects (see Figure 1). Sulindac sulphone, an NSAID that inhibits neither Cox-1 nor Cox-2, inhibits tumour formation in mice models (Piazza *et al*, 1997b). In fibroblasts, neither Cox isoform is required for malignant transformation by the oncogene ras or the SV40 virus (Zhang *et al*, 1999). In addition, celecoxib appears a more promising anti-cancer agent than rofecoxib, despite rofecoxib being a more potent inhibitor of Cox-2. Celecoxib has a sulphonamide and 4-methylphenyl moiety that may allow it to interact with other important target proteins, such as the cell-cycle regulator protein kinase B (Grosch *et al*, 2006). A potential Cox-independent intracellular target for aspirin is the transcription factor nuclear factor κb (NFκB). Aspirin inhibits the activation of NFκB (Kopp and Ghosh, 1994); this effect has been demonstrated *in vitro* and *in vivo* and is accompanied by an increase in apoptotic cells in neoplastic epithelial cells but not in normal intestinal mucosa (Stark *et al*, 2007). *In vitro* evidence also demonstrates that aspirin can potentially interact directly with other molecules and pathways implicated in tumourigenesis, including B-catenin and wnt signalling, tumour necrosis factor, polyamine metabolism and the DNA mismatch repair system (Martinez *et al*, 2003; Jankowski and Anderson, 2004; Elwood *et al*, 2009).

Apoptosis and angiogenesis are considered important physiological processes in the growth, development and treatment of cancer. Some *in vitro* studies report that aspirin is relatively inactive as an inducer of cell-cycle arrest and apoptosis and concentrations of 1 mM are required in short-term growth assays. Others found that long-term (25 days) exposure to 100–200 *μ*M of aspirin results in marked growth inhibition and argue that this a more clinically relevant model (Elder and Paraskeva, 1999). Questions as to whether aspirin-induced apoptosis is mediated through Cox inhibition are raised by observations that NSAIDs that inhibit neither Cox-1 or Cox-2 induce apoptosis and, that low-dose salicylates inhibit apoptosis *in vitro* possibly by direct effects on apoptosis-regulating genes such as *Bcl2* and *Bax* (Elwood *et al*, 2009). In addition, 2,5-dimethyl celecoxib, a structural analogue of celecoxib that does not inhibit Cox-2, induces apoptosis both *in vitro* and *in vivo*. This has been attributed to downregulation of survivin, an anti-apoptotic protein that inhibits caspase activity and increases apoptosis (Pyrko *et al*, 2006).

As early as 1983, aspirin was shown to inhibit tumour growth and vascularisation in tumours transplanted in rats (Peterson, 1983). More recently, it has been shown that aspirin at a therapeutic dose (0.5 mM) inhibits endothelial cell tubule formation, which is essential for vessel remodelling during angiogenesis. Selective inhibitors of Cox-1 and Cox-2 did not inhibit angiogenesis in this assay, suggesting that aspirin may directly inhibit angiogenesis through a Cox-independent pathway (Borthwick *et al*, 2006).

## Aspirin as an anti-cancer agent: clinical evidence

### Case–control and cohort studies: primary prevention

The first epidemiological evidence that aspirin could act as a chemoprevention agent was the report by Kune *et al* (1988) of a case–control study, in which aspirin use was associated with a significantly lower risk of colorectal cancer even after adjustment for other risk factors. In 2005, Bosetti *et al* reviewed all case–control and cohort studies up to that date, ∼100 studies, in which the use of aspirin and cancer risk was examined. The pooled relative risk (RR) for developing colorectal cancer was 0.71 (95% CI: 0.67–0.75), although there was significant heterogeneity between trials and study designs. There was more limited evidence that aspirin prevented cancers of the oesophagus (RR 0.72, 95% CI: 0.62–0.84), stomach (RR 0.84, 95% CI: 0.76–0.93), breast (RR 0.91, 95% CI: 0.88–0.95) and lung (RR 0.94, 95% CI: 0.89–1.00) (Bosetti *et al*, 2006). Two recent large cohorts have highlighted that response appeared dependent on both the duration of aspirin use and dose, with the maximum reduction in colorectal cancer incidence seen when more than fourteen 325 mg tablets were taken per week for 6–10 years (Chan *et al*, 2005, 2008).

### Randomised studies: primary prevention

**Figure 2:**
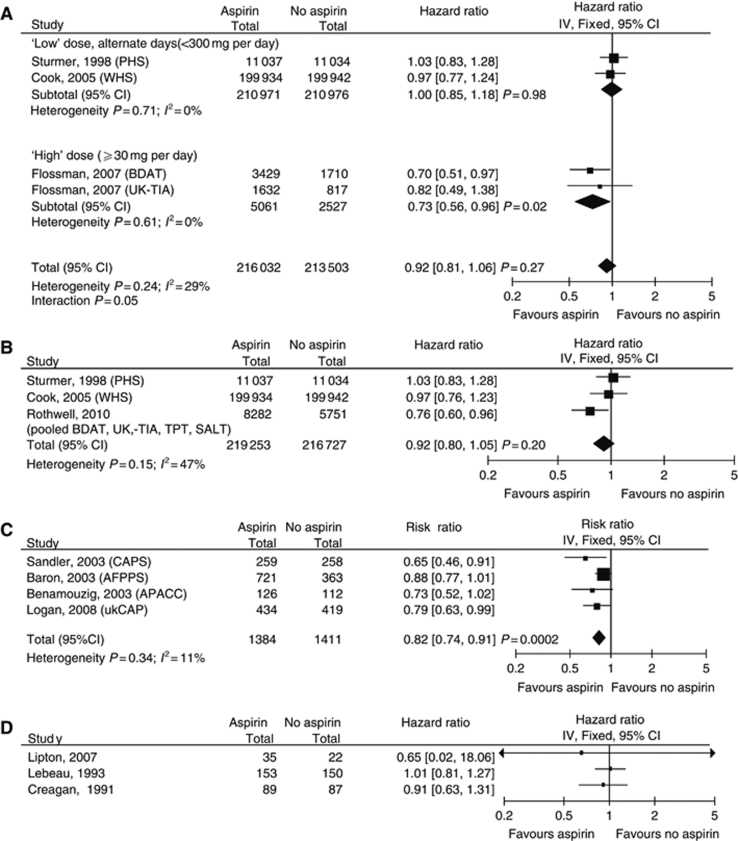
(**A**–**C**) Randomised trials of aspirin *vs* no aspirin/placebo in which colorectal cancer outcomes are available. (**A**) Includes trials designed to assess aspirin as a primary prevention agent against cancer and the first evidence from the long-term follow-up of trials primarily designed to improve cardiovascular outcomes. (**B**) Includes recent data from a meta-analysis of cardiovascular trials from which cancer incidence data were obtained. (**C**) Trials designed as secondary prevention against colorectal cancer. (**D**) Trials in which aspirin was used as a therapeutic agent against cancer with overall survival as the primary outcome measure. Details of the trials are given in Table 1.

Two large placebo-controlled trials designed to evaluate low-dose aspirin (100 or 325 mg on alternate days) as a primary prevention strategy against cancer, with a mean follow-up of ∼10 years, did not show a reduction in the risk of developing colorectal cancer (Sturmer *et al*, 1998; Cook *et al*, 2005) (Table 1 and Figure 2A). Potential reasons why these trials were negative include lack of efficacy, ineffective dose and scheduling, poor compliance and the need for even longer follow-up. Two smaller randomised trials with longer-term (median 23 years) follow-up evaluating whether higher doses of aspirin (300–1200 mg) decreased the incidence of vascular events (Flossmann and Rothwell, 2007) did show a reduction in colorectal cancer incidence (HR 0.73, 95% CI: 0.56–0.96, *P*=0.02) (Table 1 and Figure 2A), and a recent pooled analysis of individual patient data (IPD) from these trials and two others primarily assessing the cardiovascular benefits of daily aspirin has shown an overall reduction in long-term incidence of colorectal cancer (HR 0.76, 95% CI: 0.6–0.96, *P*=0.02) (Rothwell *et al*, 2010). Analysing all the randomised data of aspirin *vs* no aspirin in which colorectal cancer incidence data are available (Figure 2B) results in an HR of 0.92 (95% CI: 0.8–1.05, *P*=0.20).

A subsequent IPD meta-analysis with death from cancer as the primary outcome measure that included seven randomised trials of aspirin for primary or secondary prevention of vascular disease (Table 1) with an average treatment period of at least 4 years (Rothwell *et al*, 2011) showed a reduction in deaths from all cancers after 5 years of follow-up (HR 0.66, 95% CI: 0.50–0.87, *P*=0.003). The latent period before an effect on deaths from oesophageal, pancreatic, brain, and lung cancers was ∼5 years, but later for stomach and prostate cancers and also colorectal cancer consistent with our current understanding of the genetic events that underlie the development and progression from adenoma to colorectal carcinoma. Benefit was seen with doses as low as 75 mg daily and the absolute reduction in 20-year risk of cancer death was 7.08% (2.42–11.74) for those aged >65 years.

### Randomised studies: secondary prevention

Four randomised trials (Table 1 and Cole *et al*, 2009) have evaluated aspirin and the development of colorectal adenomas in patients previously diagnosed with colorectal cancer or adenoma. In one study, a reduction in the risk of developing further adenomas was seen counter-intuitively with low-dose aspirin (81 mg daily) (RR 0.81, 95% CI: 0.69–0.96) but not with higher doses (325 mg daily) (RR 0.96, 95% CI: 0.81–1.13, *P*=0.06) (Baron *et al*, 2003). Combining results of the secondary prevention trials in a meta-analysis (Figure 2C and Cole *et al*, 2009), suggests that aspirin reduces the RR of further adenomas by 18% (RR 0.82, 95% CI: 0.74–0.91, *P*=0.0002), with similar estimates for doses <300 mg (RR 0.82, 95% CI: 0.70–0.95, *P*=0.007) or >300 mg (RR 0.84, 95% CI: 0.74–0.94, *P*=0.004) of aspirin daily.

### Therapeutic studies

Two recent non-randomised studies have examined the use of aspirin after a diagnosis of cancer. Chan *et al* reported that compared with non-users, regular users of aspirin after a diagnosis of colorectal cancer had reduced colorectal cancer-specific mortality (HR 0.71, 95% CI: 0.53–0.95) and overall mortality (HR 0.79, 95% CI: 0.65–0.97) in a multivariate analysis. Importantly, participants whose primary tumours overexpressed Cox-2 had most benefit with an HR of 0.39 (95% CI: 0.20–0.76) for colorectal cancer-specific mortality compared with an HR of 1.22 (95% CI: 0.36–4.18) for those whose primary tumours had weak or absent Cox-2 expression. In addition, those who had taken aspirin before diagnosis did not seem to benefit (HR 0.89, 95% CI: 0.59–1.35) compared with those with no previous use (HR 0.53, 95% CI: 0.51–0.92) (Chan *et al*, 2009). Similar results have been seen for breast cancer, with aspirin use after breast cancer diagnosis associated with decreased distant recurrence and breast cancer mortality (Holmes *et al*, 2010). The adjusted RRs for 2–5 or 6–7 days of aspirin use on breast cancer mortality compared with no use were 0.29 (95% CI: 0.16–0.52) and 0.36 (95% CI: 0.24–0.65), respectively.

Three small randomised-controlled trials have assessed the effects of aspirin in combination with traditional anti-cancer therapies (Table 1 and Figure 2D). Three hundred small cell lung cancer patients were assigned to aspirin 1 g per day for 18 months or no aspirin in addition to their chemotherapy (Lebeau *et al*, 1993). There was no evidence that survival was different (HR 1.01, 95% CI: 0.81–1.27, *P*=0.09) and aspirin appeared to be well tolerated. Another trial found no evidence of a survival benefit (HR 0.91, 95% CI: 0.63–1.31, *P*=0.60) when 176 patients with advanced renal cell cancer received interferon-*α* with or without aspirin 2400 mg daily (Creagan *et al*, 1991). A small trial of only 66 patients evaluated 1200 mg of aspirin daily compared with placebo as adjuvant treatment for Duke's B2 and C colorectal cancer (HR for survival 0.65, 95% CI: 0.02–18.06, *P*=0.90) (Lipton *et al*, 1982).

## Discussion

Current drug development work recognises that the growth of tumours involves cross-talk between different signalling pathways, and that resistance develops to agents that have a single target. Aspirin affects multiple intracellular pathways and influences physiological processes such as apoptosis and angiogenesis that are important in the growth and development of malignancies (Figure 1). Publicly funded researchers have a responsibility to ensure that drugs for which there is no longer a financial incentive for pharmaceutical companies to develop further are assessed in light of current knowledge and evolving clinical practice. Aspirin pre-dates current anti-cancer strategies such as the use of adjuvant chemotherapy after a potentially curative operation. Although significant tumour shrinkage is not seen when aspirin is administered for other clinical indications such as cardiovascular disease, epidemiological evidence and pre-clinical data suggest that aspirin is worthy of further investigation particularly in the adjuvant setting, after potentially curative surgery and chemotherapy if appropriate, when disease burden is expected to be minimal.

Regular aspirin use is not currently recommended as a primary prevention strategy against cancer for those at average risk because of the risk of toxicity, particularly serious gastrointestinal bleeding. It is estimated that regular aspirin use increases the risk of a significant bleed from 1% over 10 years to 2–3% (Cuzick *et al*, 2009) and this outweighs the potential cancer benefits particularly if effective screening is available. For aspirin administered adjuvantly, the benefit:risk ratio will be different, as higher morbidity and mortality from recurrent cancer may outweigh the toxicity associated with regular aspirin use. There is also potential for wider health benefits; colorectal cancer shares similar risk factors, such as smoking and the metabolic syndrome, with coronary artery disease; thus, aspirin could potentially be beneficial from both an oncological and cardiological perspective (Chan *et al*, 2007). In any future trials the challenge will be to identify and exclude those individuals most at risk of toxicity, for example, those with a previous history of gastric ulceration (Patrono *et al*, 2001) and include those most likely to benefit. Commencing aspirin while conventional adjuvant cytotoxic chemotherapy is being administered could increase toxicity, particularly the risk of bleeding if thrombocytopaenia was present. Waiting until chemotherapy had finished would allow the use of a ‘run-in’ period, as used in adenoma prevention trials, in which a dose of 300 mg daily appeared to be well tolerated and participants were assessed as to whether they would be able to tolerate aspirin before they were randomised (Baron *et al*, 2003; Sandler *et al*, 2003). This increased compliance and reduced the risk of serious adverse events particularly gastrointestinal haemorrhage.

With the exception of the recent data from the Thrombosis Prevention Trial and the Swedish Aspirin Low Dose Trial presented by Rothwell *et al* (2010), the epidemiological data and the randomised trials assessing primary prevention support the premise that the anti-cancer effects of aspirin are most likely to be seen when higher doses are administered, there is long-term use (many years), longer follow-up (>10 years in some instances) and daily usage rather than alternate day scheduling. At higher doses, aspirin is a more potent inhibitor of Cox-2 providing a potential mechanistic explanation for these findings. The observation that the benefit of aspirin after colorectal diagnosis was greatest in those whose tumours overexpressed Cox-2, and that those who had taken aspirin before diagnosis did not appear to benefit from taking aspirin adjuvantly (Rothwell *et al*, 2010) gives an indication as to who may benefit from aspirin after a cancer diagnosis and emphasises the need for pathological assessment of tumour samples to be built into any randomised trials.

The current limited testing of aspirin (http://clinicaltrials.gov) as a therapeutic agent either in the adjuvant setting (ASCOLT and Big A trial) or in combination with other anti-cancer agents is in marked contrast to the number of studies that were initiated using selective Cox-2 inhibitors before 2004. There were numerous phase II studies and at least 12 randomised phase III trials that were ongoing in 2004, before the concerns about cardiovascular toxicity were raised, with >9000 planned participants including those with breast, colorectal, oesophageal, prostate and lung malignancies. A number of these studies involved rofecoxib and had to be discontinued when the product was withdrawn. Others were stopped early although the investigational agent (usually celecoxib) was not withdrawn.

### Conclusions

Aspirin continues to be evaluated *in vitro* and in pre-clinical models to help elucidate mechanisms involved in carcinogenesis and the response of tumours to anti-neoplastic agents. Recent randomised evidence from trials primarily designed to prevent cardiovascular disease show a reduction in cancer incidence with long-term follow-up and epidemiological evidence from colorectal and breast cancer studies evaluating the effects of aspirin use after diagnosis suggests that aspirin may have a role in the adjuvant setting. The clinical management of patients is also continually evolving, with new combinations of agents or strategies being assessed; aspirin should not be overlooked in this process because it is neither new nor expensive.

## Figures and Tables

**Table 1:** Randomised trials of aspirin *vs* no aspirin/placebo assessing cancer outcomes

**Trial**	**Accrual period**	**Participants randomised**	**Type of participants/main aim**	**Aspirin comparison**	**Aspirin duration**	**Follow-up**
*Primary prevention of cancer*						
Physicians’ Health Study (PHS) (Sturmer *et al*, 1998)	1982–1988	22 071	Male physicians 40–84 years Primary prevention of cancer	Aspirin 325 mg alternate days *vs* placebo	7–11 years	Mean=12 years
						
Women’s Health Study (WHS) (Cook *et al*, 2005)	1993–1996	39 876	Female health-care professionals ⩾45 years Primary prevention of cancer	Aspirin 100 mg alternate days *vs* placebo	5 years	Mean=10 years
						
British Doctors Aspirin Trial (BDAT) (Flossmann and Rothwell, 2007)	1978–1984	5139	UK resident male doctors <80 years Primary prevention of cardiovascular (CV) events	Aspirin 500 mg *vs* no aspirin	5–6 years	Median=23 years
						
UK Transient Ischaemic Attack trial (UK-TIA) (Flossmann and Rothwell, 2007)	1979–1985	2449	TIA or minor ischaemic stroke within 3 months, >40years Secondary prevention of CV events	Aspirin 300 or 1200 mg daily *vs* placebo	Median 4 years, range 1–7 years	Median=23 years
						
Swedish Aspirin Low-dose Trial (SALT) (Rothwell *et al*, 2010)	1984–1989	1360	Patients 50–79 years, with recent CV event or retinal artery occlusion Secondary prevention of CV events	Aspirin 75 mg daily *vs* placebo	Median 2.7 years	Until 2007 – ∼20 years from randomisation
						
Swedish Angina Pectoris Aspirin Trial (SAPAT) (Rothwell *et al*, 2011)	1985–1989	2035	Patients with chronic stable angina Primary prevention of myocardial infarction	Aspirin 75 mg daily *vs* placebo	Median 4.2 years, range 1.9–6.3 years	Until 1991
						
Thrombosis Prevention Trial (TPT) (Rothwell *et al*, 2010)	1989–1992	5085	Males 45–69 years, at high risk of CV disease Primary prevention of CV events	Aspirin 75 mg daily *vs* placebo	Median 6.9 years, range 4.3–8.6 years	Until 2009 – ∼20 years from randomisation
						
Early Treatment Diabetic Retinopathic Study (ETDRS) (Rothwell *et al*, 2011)	1980–1985	3771	Patients, 18–70 years, with diabetic retinopathy Primary prevention of death, CV events and kidney disease	Aspirin 650 mg daily *vs* placebo	Median 5 years, range 4–9 years	Mean=5 years
						
Prevention of Progression of Arterial Disease and Diabetes Trial (POPADAD) (Rothwell *et al*, 2011)	1997–2001	1276	Patients ⩾40 years, with type I/II diabetes and asymptomatic peripheral arterial disease Primary prevention of CV events	Aspirin 100 mg daily *vs* placebo	Median 6.7 years, range 4.5–8.6 years	Until 2006
						
Japanese Primary Prevention of Atherosclerosis Trial (JPAD) (Rothwell *et al*, 2011)	2002–2005	2539	Patients 30–85 years, with type II diabetes mellitus Primary prevention of CV events	Aspirin 81 or 100 mg *vs* placebo	Median 4.4 years, range 3.0–5.4 years	Until 2008
						
Aspirin for Asymptomatic Atherosclerosis Trial (AAA) (Rothwell *et al*, 2011)	1998–2008	3350	Patients 50–75 years, with low ankle brachial index and no clinical CV disease Primary prevention of CV events	Aspirin 100 mg *vs* placebo	Median 8.2 years, range 6.7–10.5 years	Mean=8.2 years
						
*Secondary prevention of cancer*						
Colorectal Adenoma Prevention Study (CAP) (Sandler *et al*, 2003)	1993–2000	635	Patients with history of colorectal cancer Secondary prevention of adenoma	Aspirin 325 mg daily *vs* placebo	3–4 years	Median=2.6 years
						
Aspirin/Folate Polyp Prevention Study (AFPPS) (Baron *et al*, 2003)	1994–1998	1121	Patients with recent history of histologically verified adenoma Secondary prevention of adenoma	Aspirin 81 or 325 mg daily *vs* placebo	A few years	Mean=2.8 years
						
Association por la Prevention par l’Aspirine du Cancer Colorectal (APACC) (Benamouzig *et al*, 2003)	1996–2000	272	Patients with history of histologically verified adenoma Secondary prevention of adenoma	Lysine acetylsalicylate 160 or 300 mg *vs* placebo	1 year	At 1 year
						
UK Colorectal Adenoma Prevention Study (ukCAP) (Logan *et al*, 2008)	1997–2001	945	Patients with recent history of adenoma Secondary prevention of adenoma	Aspirin 300 mg daily *vs* placebo	3.5–5.5 years	At 3 years
						
*Treatment of cancer*						
Lipton *et al* (1982)	Not stated	66	Patients with resected Dukes’ B2 or C colorectal cancer Cancer therapy	Aspirin 1200 mg daily *vs* placebo	2 years	Median=2 years (aspirin) Median=2.3 years (control)
						
Lebeau *et al* (1993)	1983–1985	320	Patients with limited or extensive small cell lung cancer Cancer therapy	CCAVP16 chemotherapy+1000 mg aspirin daily *vs* CCAVP16 chemotherapy	18 months	5–7 years
						
Creagan *et al* (1991)	1988–1990	179	Patients with renal adenocarcinoma Cancer therapy	IFN-*α*2A+2400 mg aspirin daily *vs* IFN-*α*2A	Not stated	Not stated

Abbreviation: IFN-*α*2A=interferon-*α*2A.

## References

[bib1] Baron JA, Cole BF, Sandler RS, Haile RW, Ahnen D, Bresalier R, McKeown-Eyssen G, Summers RW, Rothstein R, Burke CA, Snover DC, Church TR, Allen JI, Beach M, Beck GJ, Bond JH, Byers T, Greenberg ER, Mandel JS, Marcon N, Mott LA, Pearson L, Saibil F, van Stolk RU (2003) A randomized trial of aspirin to prevent colorectal adenomas. N Engl J Med 348: 891–899 12621133 10.1056/NEJMoa021735

[bib2] Benamouzig R, Deyra J, Martin A, Girard B, Jullian E, Piednoir B, Couturier D, Coste T, Little J, Chaussade S (2003) Daily soluble aspirin and prevention of colorectal adenoma recurrence: one-year results of the APACC trial. Gastroenterology 125: 328–336 12891533 10.1016/s0016-5085(03)00887-4

[bib3] Borthwick GM, Johnson AS, Partington M, Burn J, Wilson R, Arthur HM (2006) Therapeutic levels of aspirin and salicylate directly inhibit a model of angiogenesis through a Cox-independent mechanism. FASEB J 20: 2009–2016 17012253 10.1096/fj.06-5987com

[bib4] Bosetti C, Gallus S, La Vecchia C (2006) Aspirin and cancer risk: an updated quantitative review to 2005. Cancer Causes Control 17: 871–888 16841255 10.1007/s10552-006-0033-7

[bib5] Bresalier RS, Sandler RS, Quan H, Bolognese JA, Oxenius B, Horgan K, Lines C, Riddell R, Morton D, Lanas A, Konstam MA, Baron JA (2005) Cardiovascular events associated with rofecoxib in a colorectal adenoma chemoprevention trial. N Engl J Med 352: 1092–1102 15713943 10.1056/NEJMoa050493

[bib6] Cha YI, DuBois RN (2007) NSAIDs and cancer prevention: targets downstream of COX-2. Annu Rev Med 58: 239–252 17100552 10.1146/annurev.med.57.121304.131253

[bib7] Chan AO, Jim MH, Lam KF, Morris JS, Siu DC, Tong T, Ng FH, Wong SY, Hui WM, Chan CK, Lai KC, Cheung TK, Chan P, Wong G, Yuen MF, Lau YK, Lee S, Szeto ML, Wong BC, Lam SK (2007) Prevalence of colorectal neoplasm among patients with newly diagnosed coronary artery disease. JAMA 298: 1412–1419 17895457 10.1001/jama.298.12.1412

[bib8] Chan AT, Giovannucci EL, Meyerhardt JA, Schernhammer ES, Curhan GC, Fuchs CS (2005) Long-term use of aspirin and nonsteroidal anti-inflammatory drugs and risk of colorectal cancer. JAMA 294: 914–923 16118381 10.1001/jama.294.8.914 PMC1550973

[bib9] Chan AT, Giovannucci EL, Meyerhardt JA, Schernhammer ES, Wu K, Fuchs CS (2008) Aspirin dose and duration of use and risk of colorectal cancer in men. Gastroenterology 134: 21–28 18005960 10.1053/j.gastro.2007.09.035 PMC2719297

[bib10] Chan AT, Ogino S, Fuchs CS (2009) Aspirin use and survival after diagnosis of colorectal cancer. JAMA 302: 649–658 19671906 10.1001/jama.2009.1112 PMC2848289

[bib11] Chulada PC, Thompson MB, Mahler JF, Doyle CM, Gaul BW, Lee C, Tiano HF, Morham SG, Smithies O, Langenbach R (2000) Genetic disruption of Ptgs-1, as well as Ptgs-2, reduces intestinal tumorigenesis in Min mice. Cancer Res 60: 4705–4708 10987272

[bib12] Cole BF, Logan RF, Halabi S, Benamouzig R, Sandler RS, Grainge MJ, Chaussade S, Baron JA (2009) Aspirin for the chemoprevention of colorectal adenomas: meta-analysis of the randomized trials. J Natl Cancer Inst 101: 256–266 19211452 10.1093/jnci/djn485 PMC5975663

[bib13] Cook NR, Lee IM, Gaziano JM, Gordon D, Ridker PM, Manson JE, Hennekens CH, Buring JE (2005) Low-dose aspirin in the primary prevention of cancer: the Women's Health Study: a randomized controlled trial. JAMA 294: 47–55 15998890 10.1001/jama.294.1.47

[bib14] Creagan ET, Twito DI, Johansson SL, Schaid DJ, Johnson PS, Flaum MA, Buroker TR, Geeraerts LH, Veeder MH, Gesme, DH, Homburger HA (1991) A randomized prospective assessment of recombinant leukocyte A human interferon with or without aspirin in advanced renal adenocarcinoma. J Clin Oncol 9: 2104–2109 1960551 10.1200/JCO.1991.9.12.2104

[bib15] Cuzick J, Otto F, Baron JA, Brown PH, Burn J, Greenwald P, Jankowski J, La Vecchia C, Meyskens F, Senn HJ, Thun M (2009) Aspirin and non-steroidal anti-inflammatory drugs for cancer prevention: an international consensus statement. Lancet Oncol 10: 501–507 19410194 10.1016/S1470-2045(09)70035-X

[bib16] Easty GC, Easty DM (1976) Prostaglandins and cancer. Cancer Treat Rev 3: 217–225 1016977 10.1016/s0305-7372(76)80011-4

[bib17] Elder DJ, Paraskeva C (1999) Induced apoptosis in the prevention of colorectal cancer by non-steroidal anti-inflammatory drugs. Apoptosis 4: 365–372 14634339 10.1023/a:1009696505108

[bib18] Elwood PC, Gallagher AM, Duthie GG, Mur LA, Morgan G (2009) Aspirin, salicylates, and cancer. Lancet 373: 1301–1309 19328542 10.1016/S0140-6736(09)60243-9

[bib19] Flossmann E, Rothwell PM (2007) Effect of aspirin on long-term risk of colorectal cancer: consistent evidence from randomised and observational studies. Lancet 369: 1603–1613 17499602 10.1016/S0140-6736(07)60747-8

[bib20] Gasic GJ, Gasic TB, Murphy S (1972) Anti-metastatic effect of aspirin. Lancet 2: 932–933 10.1016/s0140-6736(72)92581-0 4116642

[bib21] Gasic GJ, Gasic TB, Stewart CC (1968) Antimetastatic effects associated with platelet reduction. Proc Natl Acad Sci USA 61: 46–52 5246932 10.1073/pnas.61.1.46 PMC285903

[bib22] Grosch S, Maier TJ, Schiffmann S, Geisslinger G (2006) Cyclooxygenase-2 (COX-2)-independent anticarcinogenic effects of selective COX-2 inhibitors. J Natl Cancer Inst 98: 736–747 16757698 10.1093/jnci/djj206

[bib23] Holmes MD, Chen WY, Li L, Hertzmark E, Spiegelman D, Hankinson SE (2010) Aspirin intake and survival after breast cancer. J Clin Oncol 28: 1467–1472 20159825 10.1200/JCO.2009.22.7918 PMC2849768

[bib24] Honn KV, Tang DG, Crissman JD (1992) Platelets and cancer metastasis: a causal relationship? Cancer Metastasis Rev 11: 325–351 1423821 10.1007/BF01307186

[bib25] Jankowski JA, Anderson M (2004) Review article: management of oesophageal adenocarcinoma – control of acid, bile and inflammation in intervention strategies for Barrett's oesophagus. Aliment Pharmacol Ther 20: 71–80; discussion 95–96 15456468 10.1111/j.1365-2036.2004.02143.x

[bib26] Kopp E, Ghosh S (1994) Inhibition of NF-kappa B by sodium salicylate and aspirin. Science 265: 956–959 8052854 10.1126/science.8052854

[bib27] Kune GA, Kune S, Watson LF (1988) Colorectal cancer risk, chronic illnesses, operations, and medications: case control results from the Melbourne Colorectal Cancer Study. Cancer Res 48: 4399–4404 3390835

[bib28] Langenbach R, Loftin CD, Lee C, Tiano H (1999) Cyclooxygenase-deficient mice. A summary of their characteristics and susceptibilities to inflammation and carcinogenesis. Ann N Y Acad Sci 889: 52–61 10668482 10.1111/j.1749-6632.1999.tb08723.x

[bib29] Lebeau B, Chastang C, Muir JF, Vincent J, Massin F, Fabre C, The “Petites Cellules” Group (1993) No effect of an antiaggregant treatment with aspirin in small cell lung cancer treated with CCAVP16 chemotherapy. Results from a randomized clinical trial of 303 patients. Cancer 71: 1741–1745 8383578 10.1002/1097-0142(19930301)71:5<1741::aid-cncr2820710507>3.0.co;2-q

[bib30] Levine L (1981) Arachidonic acid transformation and tumor production. Adv Cancer Res 35: 49–79 7041540 10.1016/s0065-230x(08)60908-2

[bib31] Lipton A, Scialla S, Harvey H, Dixon R, Gordon R, Hamilton R, Ramsey H, Weltz M, Heckard R, White D (1982) Adjuvant antiplatelet therapy with aspirin in colo-rectal cancer. J Med 13: 419–429 6963332

[bib32] Logan RFA, Grainge MJ, Shepherd VC, Armintage NC, Muir KR, on behalf of the ukCAP Trial Group (2008) Aspirin and folic acid for the prevention of recurrent colorectal adenomas. Gastroenterology 134: 29–38 18022173 10.1053/j.gastro.2007.10.014

[bib33] Martinez ME, O’Brien TG, Fultz KE, Babbar N, Yerushalmi H, Qu N, Guo Y, Boorman D, Einspahr J, Alberts DS, Gerner EW (2003) Pronounced reduction in adenoma recurrence associated with aspirin use and a polymorphism in the ornithine decarboxylase gene. Proc Natl Acad Sci USA 100: 7859–7864 12810952 10.1073/pnas.1332465100 PMC164678

[bib34] Patrono C, Patrignani P, Garcia Rodriguez LA (2001) Cyclooxygenase-selective inhibition of prostanoid formation: transducing biochemical selectivity into clinical read-outs. J Clin Invest 108: 7–13 11435450 10.1172/JCI13418 PMC209347

[bib35] Peterson HI (1983) Effects of prostaglandin synthesis inhibitors on tumor growth and vascularization. Experimental studies in the rat. Invasion Metastasis 3: 151–159 6203870

[bib36] Piazza GA, Alberts DS, Hixson LJ, Paranka NS, Li H, Finn T, Bogert C, Guillen JM, Brendel K, Gross PH, Sperl G, Ritchie J, Burt RW, Ellsworth L, Ahnen DJ, Pamukcu R (1997b) Sulindac sulfone inhibits azoxymethane-induced colon carcinogenesis in rats without reducing prostaglandin levels. Cancer Res 57: 2909–2915 9230200

[bib37] Powles TJ, Clark SA, Easty DM, Easty GC, Neville AM (1973) The inhibition by aspirin and indomethacin of osteolytic tumor deposits and hypercalcaemia in rats with Walker tumour, and its possible application to human breast cancer. Br J Cancer 28: 316–321 4759939 10.1038/bjc.1973.154 PMC2008894

[bib38] Pyrko P, Soriano N, Kardosh A, Liu YT, Uddin J, Petasis NA, Hofman FM, Chen CS, Chen TC, Schonthal AH (2006) Downregulation of survivin expression and concomitant induction of apoptosis by celecoxib and its non-cyclooxygenase-2-inhibitory analog, dimethyl-celecoxib (DMC), in tumor cells *in vitro* and *in vivo*. Mol Cancer 5: 19 16707021 10.1186/1476-4598-5-19 PMC1479836

[bib39] Rothwell PM, Fowkes FGR, Belch JFF, Ogawa H, Warlow CP, Meade TW (2011) Effect of daily aspirin on long-term risk of death due to cancer: analysis of individual patient data from randomised trials. Lancet 377: 31–41 21144578 10.1016/S0140-6736(10)62110-1

[bib40] Rothwell PM, Wilson M, Elwin C-E, Norrving B, Algra A, Warlow CP, Meade TW (2010) Long-term effect of aspirin on colorectal cancer incidence and mortality: 20-year follow-up of five randomised trials. Lancet 376: 1741–1750 20970847 10.1016/S0140-6736(10)61543-7

[bib41] Sandler RS, Halabi S, Baron JA, Budinger S, Paskett E, Keresztes R, Petrelli N, Pipas JM, Karp DD, Loprinzi CL, Steinbach G, Schilsky R (2003) A randomized trial of aspirin to prevent colorectal adenomas in patients with previous colorectal cancer. N Engl J Med 348: 883–890 12621132 10.1056/NEJMoa021633

[bib42] Simmons DL, Botting RM, Hla T (2004) Cyclooxygenase isozymes: the biology of prostaglandin synthesis and inhibition. Pharmacol Rev 56: 387–437 15317910 10.1124/pr.56.3.3

[bib43] Stark LA, Reid K, Sansom OJ, Din FV, Guichard S, Mayer I, Jodrell DI, Clarke AR, Dunlop MG (2007) Aspirin activates the NF-kappaB signalling pathway and induces apoptosis in intestinal neoplasia in two *in vivo* models of human colorectal cancer. Carcinogenesis 28: 968–976 17132819 10.1093/carcin/bgl220

[bib44] Sturmer T, Glynn RJ, Lee IM, Manson JE, Buring JE, Hennekens CH (1998) Aspirin use and colorectal cancer: post-trial follow-up data from the Physicians’ Health Study. Ann Intern Med 128: 713–720 9556464 10.7326/0003-4819-128-9-199805010-00003

[bib45] Takeda H, Sonoshita M, Oshima H, Sugihara K, Chulada PC, Langenbach R, Oshima M, Taketo MM (2003) Cooperation of cyclooxygenase 1 and cyclooxygenase 2 in intestinal polyposis. Cancer Res 63: 4872–4877 12941808

[bib46] Tiano HF, Loftin CD, Akunda J, Lee CA, Spalding J, Sessoms A, Dunson DB, Rogan EG, Morham SG, Smart RC, Langenbach R (2002) Deficiency of either cyclooxygenase (COX)-1 or COX-2 alters epidermal differentiation and reduces mouse skin tumorigenesis. Cancer Res 62: 3395–3401 12067981

[bib47] Ulrich CM, Bigler J, Potter JD (2006) Non-steroidal anti-inflammatory drugs for cancer prevention: promise, perils and pharmacogenetics. Nat Rev Cancer 6: 130–140 16491072 10.1038/nrc1801

[bib48] Zhang X, Morham SG, Langenbach R, Young DA (1999) Malignant transformation and antineoplastic actions of nonsteroidal antiinflammatory drugs (NSAIDs) on cyclooxygenase-null embryo fibroblasts. J Exp Med 190: 451–459 10449516 10.1084/jem.190.4.451 PMC2195603

